# Intraspecific variation and plasticity in mitochondrial oxygen binding affinity as a response to environmental temperature

**DOI:** 10.1038/s41598-017-16598-6

**Published:** 2017-11-24

**Authors:** Dillon J. Chung, P. R. Morrison, H. J. Bryant, E. Jung, C. J. Brauner, P. M. Schulte

**Affiliations:** Department of Zoology, University of British Columbia, Vancouver, BC V6T1Z4 Canada

## Abstract

Mitochondrial function has been suggested to underlie constraints on whole-organism aerobic performance and associated hypoxia and thermal tolerance limits, but most studies have focused on measures of maximum mitochondrial capacity. Here we investigated whether variation in mitochondrial oxygen kinetics could contribute to local adaptation and plasticity in response to temperature using two subspecies of the Atlantic killifish (*Fundulus heteroclitus*) acclimated to a range of temperatures (5, 15, and 33 °C). The southern subspecies of *F. heteroclitus*, which has superior thermal and hypoxia tolerances compared to the northern subspecies, exhibited lower mitochondrial O_2_ P50 (higher O_2_ affinity). Acclimation to thermal extremes (5 or 33 °C) altered mitochondrial O_2_ P50 in both subspecies consistent with the effects of thermal acclimation on whole-organism thermal tolerance limits. We also examined differences between subspecies and thermal acclimation effects on whole-blood Hb O_2_-P50 to assess whether variation in oxygen delivery is involved in these responses. In contrast to the clear differences between subspecies in mitochondrial O_2_-P50 there were no differences in whole-blood Hb-O_2_ P50 between subspecies. Taken together these findings support a general role for mitochondrial oxygen kinetics in differentiating whole-organism aerobic performance and thus in influencing species responses to environmental change.

## Introduction

Both ambient temperature and O_2_ availability vary widely across the biosphere and this has profound implications for the geographic distributions of aquatic organisms^1,2^. The physiological constraints imposed by hypoxia and temperature are thought to be a consequence of their effects on aerobic metabolism and, by extension, mitochondrial function^2–6^. Attempts to link whole organism thermal and hypoxia performance and mitochondrial function have typically examined maximum mitochondrial capacity. Studies of this nature have had mixed success in identifying mitochondrial processes that putatively constrain whole-organism performance or that are altered following environmental stress^7–17^. In addition, *in vitro* studies of maximum mitochondrial capacity can be difficult to link back to *in vivo* function because mitochondria are unlikely to operate at maximum flux *in vivo* under most circumstances^10^.

One factor that can constrain mitochondrial flux is low O_2_ supply. The effects of O_2_ supply on mitochondrial function can be described using mitochondrial O_2_ P50 (Mito-P50), the O_2_ partial pressure (PO_2_) at which mitochondrial O_2_ consumption rate is 50% of maximum flux^18^. Because Mito-P50 is measured during the aerobic to anoxic transition, it provides information on mitochondrial function over a PO_2_ range that is relevant to *in vivo* performance. In theory, a low Mito-P50 results in a greater capacity to extract O_2_ from the cytosol. Consequently, there has been interest in this parameter as a predictor of whole-organism performance. Indeed, intraspecific variation in Mito-P50 is a predictor of basal metabolic rate in humans^19^. Moreover, Lau *et al*.^19^ provide evidence for putative adaptation of Mito-P50 as a mechanism underlying variation in hypoxia tolerance among intertidal sculpin species (family: Cottidae). Links between Mito-P50 and hypoxia tolerance are not universal, however, as Du *et al*.^7^ did not observe altered Mito-P50 following hypoxic acclimation in *Fundulus heteroclitus*. By comparison, nothing is known about variation in Mito-P50 in the context of putative local thermal adaptation or thermal acclimation. It has been suggested that there may be a link between whole-organism thermal performance and systemic hypoxemia, although there is some debate about the role of systemic O_2_ limitation as a general mechanism underlying thermal tolerance limits in fishes^20,21^. However, given the relationship between ambient temperature and aerobic metabolism, prolonged thermal stress likely alters mitochondrial function and thus an investigation of temperature effects on Mito-P50 is necessary^3,4,6^.

To address this question, here we utilize *F. heteroclitus*, a eurythermal teleost found in estuarine salt marshes along a large latitudinal range that spans a steep thermal gradient [(Northern Florida, USA (mean monthly southern temperature range Sapelo Island, GA, USA: 11–30 °C^22^) to Nova Scotia, Canada (mean monthly northern temperature range 3–11 °C Wells Inlet, ME, USA^22^)]. This species is highly tolerant of both thermal and hypoxic stress, experiencing considerable variation in these abiotic factors over diel as well as seasonal cycles^23–25^. Furthermore, northern and southern *F. heteroclitus* subspecies exhibit variation in thermal and hypoxia tolerance that is consistent with apparent adaptation to their local environments^24,25^. This species recruits a wide array of physiological responses to thermal stress, including altered mitochondrial function^12,15,25–27^. Thus, this species is an ideal model in which to investigate the potential roles of mitochondrial kinetic properties in thermal acclimation and adaptation.

The objectives of this study were three-fold, (1) determine if intraspecific variation in Mito-P50 exists between putatively thermally adapted northern (*Fundulus heteroclitus heteroclitus*) and southern (*Fundulus heteroclitus macrolepidotus*) Atlantic killifish subspecies, (2) investigate the effects of thermal acclimation (5, 15, and 33 °C) on Mito-P50 and, (3) characterize the acute thermal response of Mito-P50 between *F. heteroclitus* subspecies acclimated to different temperatures. We also assessed intraspecific variation and thermal acclimation effects on hemoglobin (Hb) O_2_-P50, as there is some evidence of intraspecific variation in this parameter in *F. heteroclitus* ^28^ and any variation in this parameter could result in changes in PO_2_ gradients between the circulatory system and the mitochondrion.

## Results

### Whole-organism hypoxia tolerance (LOE_hyp_)

**Figure 1:**
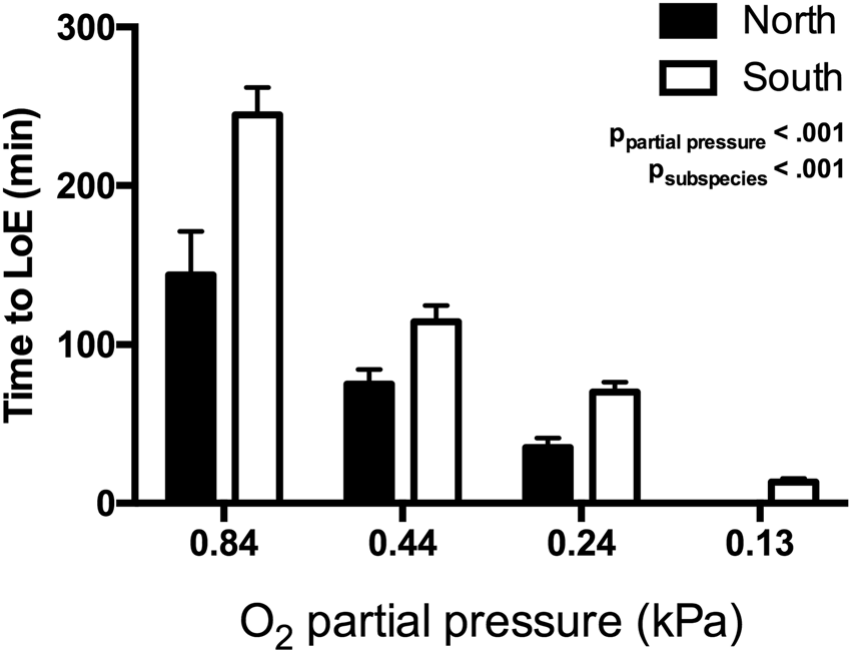
Intraspecific differences in whole-organism hypoxia tolerance. Northern and southern *F. heteroclitus* were acclimated to and assayed at 15 °C. Hypoxia tolerance was estimated using a time to loss of equilibrium assay across a range of low O_2_ partial pressures. Data are mean ± SEM, *n* = 9–10.

We measured time to loss of equilibrium in hypoxia (LOE_hyp_) at 15 °C in fish acclimated to 15 °C to confirm subspecies differences in whole organism hypoxia tolerance^25^. The northern and southern subspecies of killifish acclimated to 15 °C differed in LOE_hyp_ at T_assay_ = 15 °C across a range of PO_2_ (Fig. 1). Southern killifish exhibited greater hypoxia tolerance, as indicated by time to LOE_hyp_, when compared to northern killifish (p_subspecies_ < 0.001). PO_2_ also affected LOE_hyp,_ which decreased with decreasing PO_2_ (p_partial pressure_ < 0.001), as did the difference between the subspecies (p_partial pressure*subspecies_ < 0.05).

### Hb and Mito-P50

In this study, we examined mitochondrial O_2_ binding affinity using isolated mitochondria from the liver because high-quality mitochondria can be isolated from this tissue in this species^15,27^. These assays were conducted using a mixture of substrates at saturating levels to be representative of working or stressed states. For Mito-P50, there were significant effects of subspecies (p_subspecies_ < 0.05), assay temperature (p_assay_ < 0.0001), thermal acclimation (p_acclimation_ < 0.0005), and the interaction between thermal acclimation and assay temperature (p_acclimation*assay_ < 0.0001) on Mito-P50. No additional significant interaction effects were detected (p_subspecies*acclimation_ = 0.181, p_subspecies*assay_ = 0.144, p_population*acclimation*assay_ = 0.273) (Supplementary Fig. S1).

In contrast, there was no significant effect of subspecies (p_subspecies_ = 0.230) on Hb-P50, but we detected significant effects of thermal acclimation (p_acclimation_ < 0.05), assay temperature (p_assay_ < 0.0001), the interaction between subspecies and assay temperature (p_subspecies*assay_ < 0.001), and the interaction between subspecies, acclimation and assay temperature (p_subspecies*acclimation*assay_ < 0.05) but no other significant interaction effects (p_subspecies*acclimation_ = 0.146, p_acclimation*assay_ = 0.071). (Supplementary Fig. S2). Hill coefficients derived from Hb-O_2_ equilibrium curves did not differ between subspecies (Supplementary Fig. S3; p_subspecies_ = 0.196) or with acclimation (p_acclimation_ < 0.181), but were significantly affected by assay temperature (p_assay_ < 0.05), and there were no significant interactions (p_subspecies*acclimation_ = 0.070, p_subspecies*assay_ = 0.634, p_acclimation*assay_ = 0.637_,_ p_subspecies*acclimation*assay_ = 0.714). Hematocrit did not differ following thermal acclimation (Supplementary Fig. S4; p_acclimation_ = 0.080), or between subspecies (p_subspecies_ = 0.992), and there were no significant interactions (p_acclimation*subspecies_ = 0.350).

Because of the complex interactive effects of subspecies, acclimation temperature, and assay temperature on both Mito-P50 and Hb-P50, we tested a series of specific hypotheses regarding the effects of individual factors on both of these parameters.

### Subspecies effects on mitochondrial and Hb-O_2_ affinity

**Figure 2:**
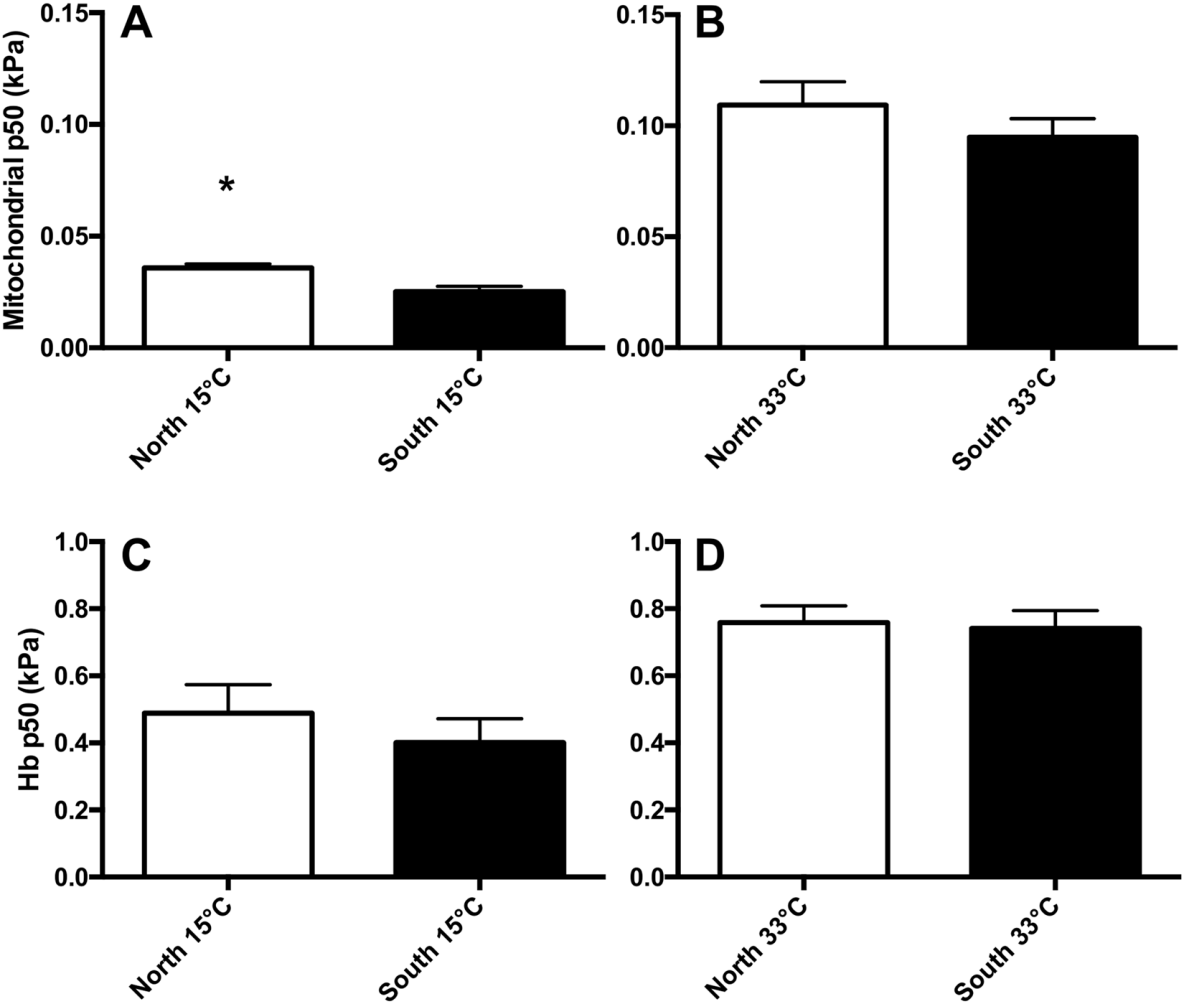
Intraspecific differences in mitochondrial. (**A**,**B**; *n* = 7–8) and hemoglobin (**C**,**D;** *n* = 7–20) O_2_ binding affinity. Northern and southern *Fundulus heteroclitus* were acclimated to 15 (**A**,**C**) or 33 °C (**B**,**D**). The assay temperature for subspecies comparisons was the same as acclimation temperature (e.g., 33 °C acclimated fish were compared at T_assay_ = 33 °C). Asterisks indicate a significant subspecies effect within an acclimation treatment (T-test, p < 0.05). Data are mean ± SEM.

We compared mitochondrial and Hb-P50 between subspecies when assayed at their acclimation temperature to test our prediction that southern killifish maintain lower O_2_ P50 when compared to northern killifish (Fig. 2). Mito-P50 was lower in southern killifish compared to northern killifish when acclimated to 15 °C and assayed at 15 °C (Fig. 2A; p_subspecies_ < 0.005), but not in 33 °C acclimated killifish assayed at 33 °C (Fig. 2B; p_subspecies_ = 0.301). Hb-O_2_ binding affinity did not differ between subspecies at either acclimation temperature (Fig. 2C,D; 15 °C: p_subspecies_ = 0.446, 33 °C: p_subspecies_ = 0.817).

### Thermal acclimation effects on mitochondrial and Hb-O_2_ affinity

**Figure 3:**
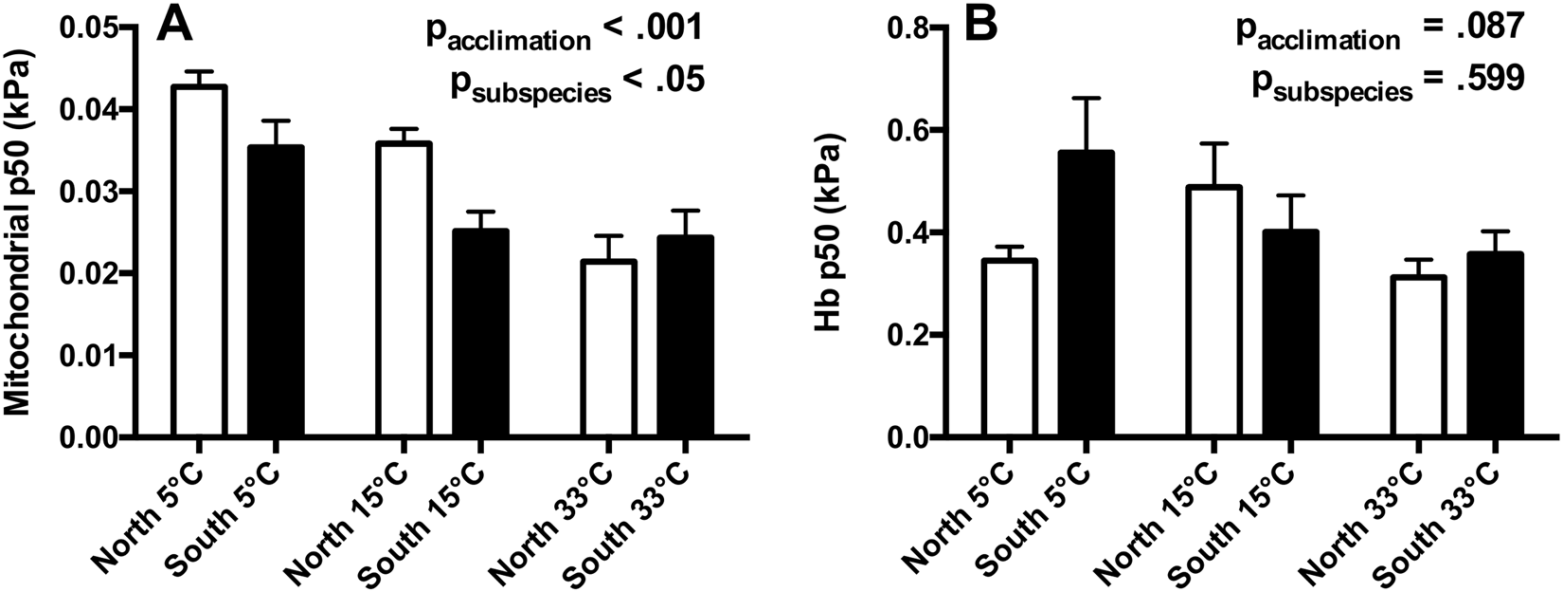
Thermal acclimation effects on mitochondrial. (**A,** *n* = 7–8) and hemoglobin (**B,** *n* = 7–20) O_2_ binding affinity. Northern and southern *Fundulus heteroclitus* were acclimated to 5, 15 or 33 °C (T_assay_ = 15 °C). Data are mean ± SEM.

We compared thermal acclimation effects (5, 15, and 33 °C) on mitochondrial (Fig. 3A) and Hb-P50 (Fig. 3B) between subspecies at a common assay temperature of 15 °C to test our prediction that acclimation to higher temperatures would result in lower O_2_ P50 for both parameters. We used 5 and 33 °C as our experimental acclimation temperatures as these are temperatures at which effects on whole-organism aerobic metabolism are first observed^29^. In addition, we acclimated *F. heteroclitus* to 33 °C to avoid the induction of substantial breeding physiology at slightly lower temperatures (i.e., 25 to 30 °C). These acclimation treatments represent the majority of temperatures experienced by natural *F. heteroclitus* populations^22^.

We detected a significant effect of acclimation on Mito-P50 (Fig. 3A; p_acclimation_ < 0.001). This effect was driven by higher Mito-P50 in 5 °C acclimated killifish and lower Mito-P50 in 33 °C acclimated northern killifish. We also detected a significant effect of subspecies that was driven by greater Mito-P50 in northern killifish (p_subspecies_ < 0.05). Significant interactions between subspecies and thermal acclimation effects were a result of subspecies differences being removed as T_acclimation_ increased to 33 °C (p_subspecies*acclimation_ < 0.05).

We did not detect a significant effect of acclimation on Hb-P50 (Fig. 3B; p_acclimation_ = 0.087) or an effect of subspecies (p_subspecies_ = 0.599) or an interaction between subspecies and acclimation temperature (p_subspecies*acclimation_ = 0.140).

### Acute thermal effects on mitochondrial and Hb-O_2_ affinity

**Figure 4:**
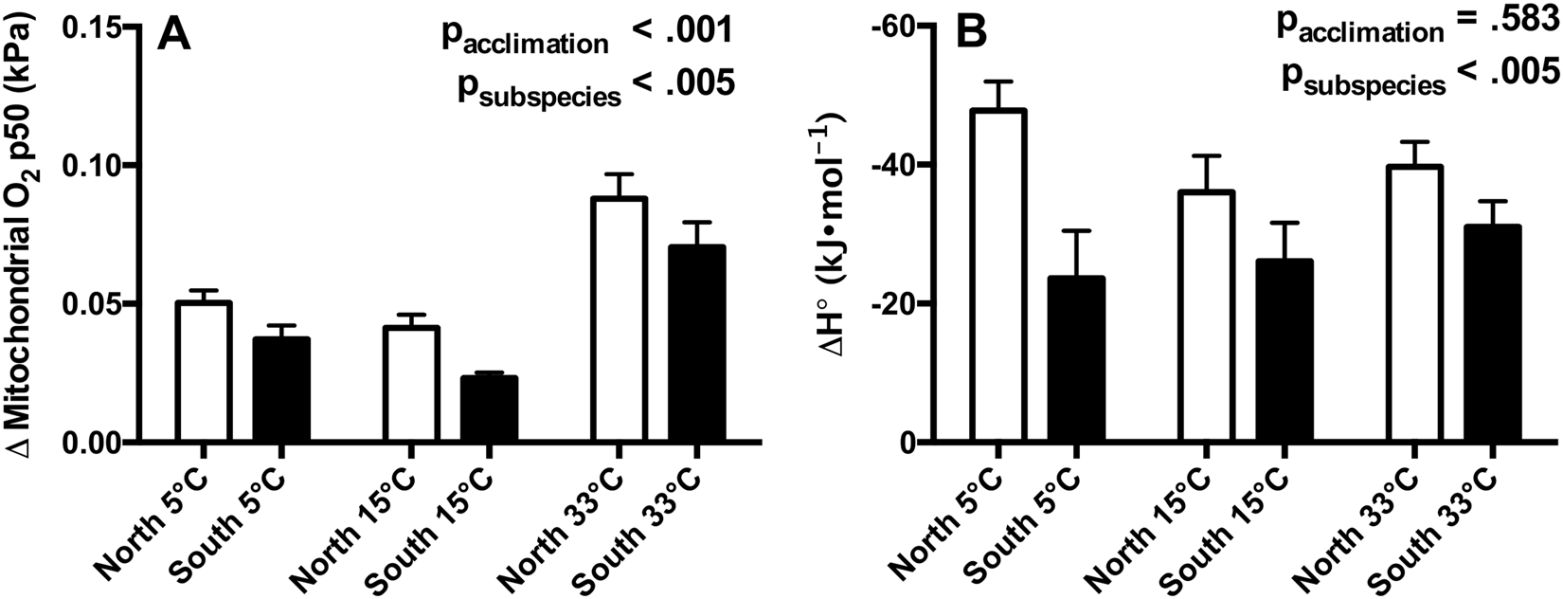
Acute thermal sensitivity (T_assay_ = 15 to 33 °C) of mitochondrial (**A**) Δ mitochondrial O_2_ P50; *n* = 7–8) and hemoglobin (**B**) ΔH°; *n* = 7–20) O_2_ P50. Northern and southern *Fundulus heteroclitus* were acclimated to 5, 15, or 33 °C. A more negative ΔH° is indicative of favorable Hb-O_2_ binding at lower temperatures. Data are mean ± SEM.

We assessed the effects of thermal acclimation and local adaptation on Δ Mito-P50 (i.e., the difference in Mito-P50 between T_assay_ = 15 and 33 °C) and the heat of oxygenation of Hb, ΔH (Fig. 4). We predicted that thermal acclimation and putative local adaptation would alter the acute thermal response for both parameters. Acute changes in temperature alter buffer pH, and this has the potential to affect both Mito-P50 and Hb-P50. But this variation in pH does not affect the interpretation of our specific predictions as most comparisons were made at the same assay temperature. The only exception is our assessment of acute thermal effects on O_2_ binding affinity (Fig. 4), which might be subject to an interaction between intraspecific variation or thermal acclimation effects and pH variation induced by the acute temperature shift between 15 and 33 °C.

Acclimation temperature affected Δ Mito-P50 (Fig. 4A; p_acclimation_ < 0.001). Northern *F. heteroclitus* exhibited a greater Δ Mito-P50 when compared to the southern subspecies (p_subspecies_ < 0.005). No significant interaction effects were detected (p_acclimation*subspecies_ = 0.907).

Thermal acclimation did not significantly change ΔH (Fig. 4B; p_acclimation_ = 0.583). Northern *F. heteroclitus* maintained more negative ΔH when compared to southern *F. heteroclitus* (p_subspecies_ < 0.005). No significant interaction effects were detected (p_acclimation*subspecies_ = 0.371).

## Discussion

Here we demonstrate that Mito-P50 differs between putatively thermally adapted subspecies of killifish and is sensitive to thermal acclimation. These effects on Mito-P50 are consistent with intraspecific variation and the effects of thermal acclimation on whole-organism thermal and hypoxia tolerance^25^. These data provide evidence for altered mitochondrial oxygen affinity as a potential mechanism for maintaining whole-organism performance under environmental stress, which may ultimately contribute to subspecies-specific differences in thermal tolerance.

There were clear intraspecific differences in Mito-P50 between northern and southern *F. heteroclitus* subspecies, with southern fish maintaining lower O_2_-P50 (Fig. 2A, S1). We propose that variation in Mito-P50 partially underlies intraspecific variation in hypoxia tolerance (Fig. 1 ^25^). Intraspecific variation in Mito-P50 may be a consequence of variation in genes encoding cytochrome *c* oxidase (CCO) subunits. CCO is the terminal acceptor of the electron transport chain and the primary site of O_2_ consumption in the mitochondrion. In addition, CCO subunits are encoded by both nuclear and mitochondrial genomes, the latter being subject to high mutation rates. Consequently, CCO function has been demonstrated to be a target of selection^30–32^. Genome sequencing efforts in *F. heteroclitus* ^9^ have revealed mixed evidence for the presence of functionally significant variation in CCO among *F. heteroclitus* populations, but at present there have been no comprehensive examinations of sequence variation between populations from the extremes of the species distribution. In addition, differences in CCO function could be a consequence of variation in mitochondrial membrane composition or post-translational modifications of the enzyme^33^.

In contrast to the variation between subspecies in Mito-P50, we did not observe significant differences in Hb-P50 between *F. heteroclitus* subspecies (Fig. 2C,D), consistent with previous observations in a hybrid *F. heteroclitus* population^28^. However, in this population individuals with differing LDH-B genotypes differed in Hb-P50 following exhaustive swimming, implicating a genotype-specific Bohr shift (i.e., low pH decreasing O_2_ affinity) due to allosteric modification of Hb by ATP^28^. These differences are thought to result from differences in glycolytic metabolism due to variation in LDH-B^34^. Both subspecies maintain equivalent hematocrit (Supplementary Fig. S4 ^28^), indicating that if Hb characteristics differentiate aerobic performance between subspecies it likely occurs through allosteric mechanisms.

Hb-P50 in killifish (approximately 0.4 kPa) is low relative to other fish species (e.g., Hb-P50 = 3.6 kPa in *Oncorhynchus mykiss* ^35^; 2.6 kPa in *Kryptolebias marmoratus* ^36^; and ranges between 3–8 kPa among intertidal sculpins^37^) suggesting that selection may have acted on *F. heteroclitus* to maximize O_2_ extraction from the environment. However, for Hb there is a trade-off between the ability to load O_2_ at the gills and unload at the tissues. The fact that we do not observe much variation between subspecies in Hb-P50 may reflect this trade-off. Thus, variation in Mito-P50 could represent an adaptation to maximize tissue O_2_ diffusion in the hypoxia tolerant southern subspecies. The hypothesis of a tissue O_2_ diffusion limitation in this species is supported by electron microscope observations of the location of the mitochondrion in this species, which at least in muscle are localized immediately below the plasma membrane^26^. However, both Hb-P50 and Mito-P50 are subject to considerable regulation *in vivo*, and this is an effect that we are unable to account for in our assays. Nevertheless, the difference in O_2_ binding affinity between Hb and the mitochondrion likely contributes to the shape of O_2_ diffusion gradients at the tissue^38^.

In humans, there is an inverse relationship between basal metabolic rate and Mito-P50^32^. We therefore predicted that northern *F. heteroclitus* would have lower Mito-P50 than southern fish, because northern populations maintain higher routine metabolic rates than do their southern counterparts^29^. In contrast, we found that the southern subspecies has a lower Mito-P50, suggesting that the relationship between metabolic rate and Mito-P50 does not represent a functional constraint in *F. heteroclitus*. Alternatively, the inconsistent relationship between Mito-P50 and estimates of basal metabolism in humans and *F. heteroclitus* may be a consequence of the energetic demands imposed by endothermy when compared with ectothermy. The combination of low routine metabolic rate and Mito-P50 exhibited by the southern subspecies may represent a beneficial strategy in high temperature hypoxic environments, allowing for greater tissue O_2_ extraction while also decreasing overall demand in the face of environmental hypoxia.

Organisms’ thermal tolerance limits are thought to be shaped by temperature effects on aerobic metabolism that may, at least in part, be due to effects at the level of the mitochondrion^3,6,13^. However, these effects have mostly been examined in the context of mitochondrial respiratory capacity. Here we show that northern and southern *F. heteroclitus* subspecies that differ in thermal tolerance^24^ also exhibit differences in Mito-P50. The lower Mito-P50 in the southern subspecies, which reflects a greater mitochondrial oxygen affinity, could aid in the maintenance of mitochondrial O_2_ diffusion gradients, particularly at high temperatures, which could help to sustain aerobic metabolism at high temperatures. Similarly, low Mito-P50 has the potential to aid O_2_ delivery during environmental hypoxia. Thus, in southern habitats where temperatures are higher and hypoxic events are more likely, low Mito-P50 could be favored. Alternatively, the relatively low temperatures and higher oxygen levels in northern habitats might result in relaxed selection on these traits, reducing the constraints on Mito-P50 in this subspecies. Taken together, our demonstration of intraspecific variation in Mito-P50 thus provides a candidate trait accounting for potential covariation of both whole-organism hypoxia and thermal tolerance.

Acclimation to both 5 and 33 °C resulted in clear changes in Mito-P50 that were subspecies-dependent (Fig. 3, S1). In contrast, Hb-P50 did not exhibit a clear response to thermal acclimation (Fig. 4, S2). This previously undescribed phenomenon identifies potential targets and mechanisms of thermal acclimation and provides support for a role for mitochondrial function in maintaining performance following prolonged thermal stress.

We predicted that acclimation to 33 °C would decrease Mito-P50 thereby compensating for the aerobic challenges associated with high temperature^3,8,15,39^. When compared at a common assay temperature of 15 °C, acclimation to 33 °C decreased Mito-P50 in northern but not southern *F. heteroclitus* (Fig. 3A). Decreases in Mito-P50 under these conditions may alleviate limitations on total O_2_ flux suggested to occur with elevated temperatures^3^ and is consistent with the maintenance of a PO_2_ gradient to the mitochondrion. In contrast, southern *F. heteroclitus* exhibit low Mito-P50 (high oxygen affinity) at both intermediate and high temperatures, indicating a difference in strategy between the subspecies. However, when compared at a higher assay temperature of 33 °C, acclimation to 33 °C increased Mito-P50 in both subspecies (Supplementary Fig. S1). This paradoxical response could represent maladaptive acclimation as this would presumably decrease O_2_ diffusion gradients to the tissues. Thus, it is possible that this response may instead be a consequence of sub-lethal effects associated with 33 °C acclimation. 33 °C is a non-lethal temperature to which *F. heteroclitus* can acclimate for prolonged periods^24^. However, acclimation to 33 °C is associated with decreases in whole body mass and routine O_2_ consumption, perhaps indicative of trade-offs necessary to mitigate the effects of extremely high acclimation temperatures on energetic balance^8,29^. Thus, our observed change in Mito-P50 may be a consequence of other mitochondrial responses associated with high temperature acclimation (e.g., altered mitochondrial morphology and membrane composition^40,41^). This raises interesting questions as to the mitochondrial responses of organisms under conditions of sub-lethal stress, which are likely to have a profound influence on species' fitness^42–44^.

In both subspecies, we observed higher Mito-P50 in fish acclimated to low temperature when assayed at 15 °C (Fig. 3A). Indeed, cold acclimation is associated with increases in mitochondrial respiratory capacity, mitochondrial volume density and lipid content in aquatic ectotherms^14,15,26,41^. Increases in lipid content increase O_2_ solubility^45^ which may alleviate potential mitochondrial O_2_ limitations in systemic tissues at low temperatures that are brought on by decreased O_2_ diffusion rates^46^. This 5 °C acclimation response may reveal a mechanism for life at low temperatures as it is consistent with the greater Mito-P50 exhibited by northern *F. heteroclitus* (Fig. 2).

Similar to our demonstration of thermal acclimation effects on Mito-P50 (Fig. 3A), prolonged exposure to hypoxia might be predicted to decrease Mito-P50^7,17^, if reductions in Mito-P50 are important for maintaining O_2_ diffusion gradients. However, hypoxia acclimated northern *F. heteroclitus* exhibit no modification of Mito-P50^7^. This suggests that declines in Mito-P50 at high temperatures are not directly mediated by the associated environmental hypoxia or resulting hypoxemia. Thus, the effects of thermal acclimation that we observe may play a role other than maintaining oxygen diffusion gradients. Alternatively, during hypoxic acclimation this species could recruit other mechanisms for maintaining O_2_ supply and demand balance, such reductions in demand^23^ would reduce the necessity for decreased Mito–P50 following acclimation.

Although we detected intraspecific variation and thermal acclimation effects on liver Mito-P50, this effect might not be present in mitochondria from other tissues^47^. Indeed, thermal acclimation and intraspecific variation effects on *F. heteroclitus* mitochondrial respiratory capacity vary among the heart, liver and brain^8,15,27^, suggesting that mitochondria from different tissues may respond differently. The role of liver mitochondria in constraining whole-organism hypoxia and thermal tolerance is not clear, whereas other tissues such as the heart and brain may be more important^3,5,21^. Consistent with this idea, variation in Mito-P50 from brain mitochondria has been shown to be associated with evolutionary variation in hypoxia tolerance^19^ whereas Mito-P50 from liver mitochondria does not change in response to hypoxic acclimation in *F. heteroclitus* despite changes in whole-organism hypoxia tolerance^7^. Thus, the role of the differences in liver Mito-P50 that we observe in setting whole organism thermal or hypoxia tolerance requires further investigation.

As assay temperature increased (assay temperature: 15–33 °C), both Hb and Mito-P50 increased (Figs S1, S2). The loss of Hb O_2_ affinity associated with increasing assay temperature is often observed and is primarily a consequence of the exothermic nature of Hb oxygenation^48^. Decreased Hb O_2_ binding affinity with acute increases in temperature might aid in unloading O_2_ to the tissues but might also compromise O_2_ loading at the gills. However, these effects are likely subject to considerable regulation *in vivo*. In contrast, decreased mitochondrial O_2_ affinity with increased assay temperature has not been previously characterized and is presumably a result of temperature effects on enzyme stability. This decrease in mitochondrial O_2_ binding affinity at high assay temperatures might cause a decline in mitochondrial ATP synthesis, perhaps revealing an aspect of declining mitochondrial function associated with acute increases in temperature.

We detected clear subspecies effects on the acute thermal sensitivity of O_2_ binding affinity at all acclimation temperatures (Fig. 3). Northern *F. heteroclitus* exhibited greater Δ Mito-P50 and a more exothermic Hb ΔH (i.e., greater thermal sensitivity) when compared to the southern subspecies (T_assay_ = 15 to 33 °C). These data indicate that northern *F. heteroclitus* exhibit a greater relative loss of O_2_ binding affinity than the southern subspecies following acute increases in temperature. This loss of O_2_ binding affinity at high acute temperatures may result in greater constraints on aerobic metabolism in this subspecies, which could be associated with the differences between subspecies in acute thermal tolerance limits^24^.

Thermal acclimation also altered the acute thermal sensitivity of Mito-P50 (Supplementary Fig. S1). This is reflected by increased Δ Mito-P50 following 33 °C acclimation in both *F. heteroclitus* subspecies (Fig. 4A). This increase in sensitivity may be a consequence of sub-lethal effects associated with 33 °C acclimation and changes in mitochondrial morphology as discussed previously. In contrast, we did not detect significant thermal acclimation effects on hemoglobin thermal sensitivity (ΔH°, Fig. 4B), although such effects have been detected in other fish species. (e.g., *Oncorhynchus mykiss* ^49^).

In this study, we demonstrate clear effects of intraspecific variation and thermal acclimation on Mito-P50. This variation in kinetic properties is consistent with subspecies differences and the effects of thermal acclimation on hypoxia and thermal tolerance. These changes in Mito P50 may help to maintain oxygen diffusion gradients particularly at high temperatures. We thus propose that altered Mito-P50 is involved in differentiating organism-level aerobic and thermal performance between putatively adapted *F. heteroclitus* subspecies and in response to thermal acclimation and could represent a novel target for thermal adaptation.

## Methods

### Animals

All procedures were carried out at the University of British Columbia according to the University of British Columbia Animal Care Committee approved protocol #A01-0180. Northern (*Fundulus heteroclitus* macrolepidotus; Ogden’s Pond Estuary, NS, 45°71′N; 61°90′W) and southern (*Fundulus heteroclitus* heteroclitus; Jekyll Island, GA, 31°02′N; 81°25′W) Atlantic killifish were collected in summer, 2014, and were transported to UBC's Aquatics Facility where they were kept in 190 L tanks with biological filtration. Fish were held at 15 ± 2 °C, and 20 ppt salinity, with a 12:12 L:D photoperiod. Animals were fed once daily to satiation (Nutrafin Max). In June 2015, fish were transferred to 114 L tanks with biological filtration. Temperature was held at 5, 15 or 33 ± 2 °C and all other conditions were maintained. After a minimum of four weeks of thermal acclimation, fish were fasted for 24 h and sampled as described below.

### Loss of equilibrium in hypoxia assay

Brackish water (T_a_ = 15 °C, 20 ppt salinity) was circulated in a 50 l plexiglass arena with bubblewrap at the water's surface to prevent O_2_ diffusion. Fish were placed in individual containers in the arena (10 fish per trial, 1 fish per container), where the former allowed for full mixing of water while preventing access to the water's surface. PO_2_ was monitored continuously using a fiber-optic oxygen probe (NEO-Fox, Ocean Optics, Dunedin, FL). Following 10 min of acclimation to the container, PO_2_ was decreased over 30 min by bubbling N_2_ gas to one of four PO_2_ values (0.84, 0.44, 0.24, or 0.13 ± .02 kPa). When the desired PO_2_ was reached, the trial began and time to LOE_hyp_ was recorded. LOE_hyp_ was determined as the time at which fish no longer responded to gentle movement of their containers following which fish were removed and placed in a recovery tank.

### Hemoglobin O_2_ P50 and hematocrit content

*Fundulus heteroclitus* were removed from the 5, 15, or 33 °C thermal acclimation tanks at 8:00 AM PST, euthanized by cervical dislocation and weighed. The caudal peduncle was severed and blood was collected from the incision using micro-hematocrit tubes. Micro-hematocrit tubes were centrifuged (10 min, 12,700 *g*, 25 °C) and hematocrit was measured as the volume percentage of packed RBCs within the total blood sample.

Micro-hematocrit tubes were separated at the boundary between RBCs and plasma. RBCs were re-suspended to approximately the same measured hematocrit content with buffered saline (50 mM HEPES with 100 mM NaCl, final osmolality = 390 Osm kg^−1^, pH = 7.8 at 25 °C) for O_2_ equilibria experiments^50^. Osmolality of the buffered saline solution was set based on plasma osmolality measurements (10 μL of whole blood measured using standard protocols with an osmometer, Vapro 5520, Wescor, Logan, UT) from five randomly selected 15 °C acclimated *F*. *heteroclitus* (396 ± 14 Osm⋅kg^−1^ mean ± sd).

Oxygen equilibrium curves were generated using protocols described by Lilly *et al*.^51^. Re-suspended RBCs (3 μL) were sandwiched between two sheets of low density polyethylene that were secured on an aluminum ring with two plastic O-rings. Blood samples were placed in a gas tight tonometry cell modified to fit into a SpectraMax 190 microplate reader (Molecular Devices, Sunnyvale, USA). Assay temperature was maintained at 15 or 33 °C, and blood from the three acclimation temperatures were assayed at both assay temperatures. Samples were equilibrated with pure N_2_ for 30 min to achieve full Hb deoxygenation (deoxyHb). O_2_ tension was increased by 6 to 12 increments of air (21% O_2_) balanced with N_2_ using a Wӧsthoff DIGAMIX gas mixing pump (H. Wösthoff Messtechnik, Bochum, Germany). Optical density (OD) was measured every 10 s at 390 nm, and 430 nm, which correspond to the isosbestic point (i.e., OD is independent of Hb-O_2_ saturation), and maxima for deoxygenated Hb, respectively. Hb was assumed to be fully saturated (oxyHb) with O_2_ (100% air) when no change in OD at 430 nm was detected after three equilibration steps.

Hb-O_2_ saturation for each equilibration step was calculated using Eqn. 1.

1 $$Hb-{O}_{2}\,saturation=\,\frac{(O{D}_{430nm}-O{D}_{390nm})}{[{(O{D}_{430nm}-O{D}_{390nm})}_{oxyHb}-{(O{D}_{430nm}-O{D}_{390nm})}_{deoxyHb}]}$$

Oxygen equilibrium curves (OECs) were constructed for each sample by non-linear least squares curve fitting to fit the Hb-O_2_ saturation data to the Hill equation (Eqn. 2).

2 $$y=\frac{P{{\rm{O}}}_{2}^{n}}{P{{\rm{O}}}_{2}^{n}+{P}_{50}^{n}}$$

Where *P50* is the *P*O_2_ at which Hb is 50% saturated and is a measure of Hb-O_2_ affinity, and *n* is the cooperativity (Hill) coefficient^51^. All curves used in the final analyses fit well to the data (r^2^ > 0.99).

We estimated the effects of acute thermal shifts (T_assay_ = 15 to 33 °C) on Hb-O_2_ affinity by calculating the apparent heat of oxygenation using the van't Hoff isochore (ΔH, Eqn. 3 ^52^).

3 $$\Delta H=2.303\cdot R\cdot \frac{{\rm{\Delta }}\,\mathrm{log}({P}_{50})}{(\frac{1}{{T}_{1}-{T}_{2}})}$$

Where R is the gas constant and T_1_ and T_2_ are the absolute temperatures 306 and 288 K respectively.

### Liver mitochondrial isolation

Seven *F. heteroclitus* were removed from the 5, 15, or 33 °C thermal acclimation tanks and euthanized for liver mitochondrial isolation as described previously^15^. Liver tissue was excised and pooled into one aliquot of ice-cold homogenization buffer (250 mM sucrose, 50 mM KCl, 0.5 mM EGTA, 25 mM KH_2_PO_4_, 10 mM HEPES, 1.5% BSA, pH = 7.4 at 20 °C). Liver tissue was cut into approximately 1 mm^3^ pieces and homogenized with 5 passes of a loose-fitting Teflon pestle followed by filtration through 1-ply cheesecloth. Crude liver homogenate was centrifuged at 4 °C for 10 min at 600 *g*. The fat layer was removed with aspiration and the remaining supernatant was filtered through 4-ply cheesecloth. Filtered supernatant was centrifuged at 4 °C for 10 min at 6000 *g*. The resulting pellet was washed twice and suspended in 800 μL of homogenization buffer and stored on ice until experimentation. Protein content was determined using a Bradford assay with BSA as a standard^53^.

### Mitochondrial assays

Mitochondrial O_2_ binding affinity (Mito-P50) was measured using a high-resolution respirometry system (O2k MiPNetAnalyzer; Oroboros Instruments, Innsbruck, Austria). Air-saturated and O_2_-depleted (achieved with a yeast suspension) calibrations of respiration buffer (MiRO5; 110 mM sucrose, 0.5 mM EGTA, 3 mM MgCl_2_, 60 mM K-lactobionate, 20 mM taurine, 10 mM KH_2_PO_4_, 20 mM HEPES, 0.1% BSA, pH = 7.1 at 30 °C^54^) were taken at each assay temperature (15, 33 and 37 °C) using published O_2_ solubilities^55^. Mito-P50 was determined using DatLab 2 software (Oroboros Instruments). Mitochondrial respiration rate during the transition into anoxia was fit against Eqn. 4.

4 $${\dot{M}}_{{O}_{2}}=\frac{{J}_{max}\times {P}_{{O}_{2}}}{{P}_{50}+{P}_{{O}_{2}}}$$

where ${\dot{M}}_{{O}_{2}}$ is the mitochondrial respiration rate, *J*_*max*_ is maximal respiration rate and *P*_50_ is the ${P}_{{O}_{2}}$ at which respiration rate is half of *J*_*max*_. We accounted for the time delay of the O_2_ sensor, background O_2_ consumption and internal zero drift at each assay temperature^18^.

Two ml of MiRO5 was air-equilibrated at each assay temperature (15, 33 and 37 °C) followed by the addition of liver mitochondria (0.5 mg protein). A saturating mixture of substrates (glutamate 10 mM, pyruvate 10 mM, malate 2 mM, succinate 10 mM, palmitoyl-carnitine 20 mM) was added to the chamber followed by ADP (2.5 mM) to fuel mitochondrial respiration. Mitochondrial respiration was allowed to proceed until all O_2_ was consumed (i.e., anoxia). Anoxic conditions were maintained for 15 min, followed by the termination of the experiment.

The effects of acute temperature shifts on Mito-P50 (i.e., Δ Mito-P50) were estimated by calculating the difference in Mito-P50 between T_assay_ = 15 and 33 °C for each treatment.

### Statistics and calculations

Statistical tests were completed using R software (v3.3.3). Data are presented as mean ± SEM and α was set at.05. We confirmed the presence of normal distributions and homogeneity of variance in our data using Shapiro-Wilk and Bartlett's tests respectively. Sample size is indicated (*n*) in the respective figure captions.

We compared the effects of subspecies, thermal acclimation, assay temperature using three-separate linear mixed effect models (individual as the random effect) to compare Hb and Mito-P50 and Hill coefficients.

We used separate t-tests to compare subspecies effects on Hb and mitochondrial O_2_ binding affinity. Comparisons were made between subspecies assayed at their acclimation temperature (e.g., 33 °C acclimated northern and southern fish assayed at 33 °C). We accounted for an increased false discovery rate by adjusting α using a Benjamini-Hochberg correction for our specific test of subspecies effects.

Thermal acclimation effects (5, 15, and 33 °C) were assessed between subspecies at T_assay_ = 15 °C using a two-way ANOVA. The effects of acute thermal shifts (T_assay_ = 15 to 33 °C) on mitochondrial (Δ Mito-P50) and Hb- P50 (ΔH) were assessed between subspecies and among thermal acclimation treatments using a two-way ANOVA.

Thermal acclimation and subspecies effects on hematocrit content were assessed using a two-way ANOVA. The effects of subspecies and decreasing PO_2_ on time to LOE_hyp_ were assessed using a two-way ANOVA.

### Data availability

The data generated during the current study are available from the corresponding author on reasonable request.

## Electronic supplementary material

Supplementary Materials 1
