# Supplementary material for: Intraspecific variation and plasticity in mitochondrial oxygen binding affinity as a response to environmental temperature
Source: Sci Rep. 2017 Nov 24;7:16238. doi: 10.1038/s41598-017-16598-6 (PMC5701142; doi:10.1038/s41598-017-16598-6)
Supplement: Supplementary file 1 — Supplementary Materials 1 [file 41598_2017_16598_MOESM1_ESM.doc]

Intraspecific variation and plasticity in mitochondrial oxygen binding affinity as a response to environmental temperature

Dillon J. Chung; Morrison, P.R.; Bryant, H.J.; Jung, E.; Brauner, C.J.; Schulte, P.M.

**Supplementary Figure S1.** Full-model of thermal acclimation and intraspecific variation effects on mitochondrial O_2_ binding affinity from *Fundulus heteroclitus*. Northern (A) and southern (B) *F. heteroclitus* were acclimated to 5 (black circle), 15 (grey square), or 33°C (white triangle) for four weeks. Data are mean ± SEM, n = 7-8.

**Supplementary Figure S2.** Full-model of thermal acclimation and intraspecific variation effects on whole blood hemoglobin O_2_ binding affinity from *Fundulus heteroclitus*. Northern (A) and southern (B) *F. heteroclitus* were acclimated to 5 (black circle), 15 (grey square), or 33°C (white triangle) for four weeks. Data are mean ± SEM, n = 7-20.

**Supplementary Figure S3.** Full-model of thermal acclimation and intraspecific variation effects on Hill coefficients derived from hemoglobin O_2_ equilibrium curves from *Fundulus heteroclitus*. Northern (A) and southern (B) *F. heteroclitus* were acclimated to 5 (black circle), 15 (grey square), or 33°C (white triangle) for four weeks. Data are mean ± SEM, n = 7-20.

**Supplementary Figure S4.** Thermal acclimation and intraspecific variation effects on hematocrit in *Fundulus heteroclitus*. Northern (white triangle) and southern (black circle) *F. heteroclitus* were acclimated to 5, 15, or 33°C for four weeks. Data are mean ± SEM, n = 7-20.
